# The Use of Re-entry Devices in Aortoiliac Occlusive Disease

**DOI:** 10.3389/fcvm.2020.00144

**Published:** 2020-08-25

**Authors:** Lorenzo C. Pescatori, Vania Tacher, Hicham Kobeiter

**Affiliations:** ^1^Assistance Publique - Hôpitaux de Paris (AP-HP), Service d'Imagerie Médicale, CHU Henri Mondor, Créteil, France; ^2^Université Paris-Est Créteil (UPEC), Créteil, France; ^3^Unité INSERM U955 #18, IMRB, Créteil, France

**Keywords:** re-entry catheter, aortoiliac occlusion, subintimal recanalization, chronic total occlusion, extraluminal

## Abstract

Endovascular approach is the first-choice treatment in patients suffering from aortoiliac occlusions. Nevertheless, standard endoluminal revascularization fails in treating occlusions in about 20% (1) of cases. Thus, subintimal revascularization can be a solution, but it fails in 25% (2) of cases as well. In the last decades, different devices have been created, in order to ease the cross back into the true lumen, when standard subintimal revascularization does not work or risks to occlude important collateral vessels. Herein, we revise the currently available re-entry devices and their application in the aortoiliac occlusive pathology.

## Introduction

Aortoiliac disease is a major cause of claudication and/or critical limb ischemia, with a prevalence up to 10% in the general population, increasing up to 20% in patients aged over 70 (1).

In 2007, the Inter-Society Consensus for the Management of Peripheral Arterial Disease (TASC II) stratified aortoiliac lesions considering length, grade of occlusion, and position and suggested endovascular (EV) approach for lesions classified TASC A and B and open surgery (OS) for lesions TASC C and D, including chronic total occlusions (CTOs) (3).

CTOs of the aortoiliac tract (AT) are challenging situations and they account for almost 35% of iliac interventions (4). In this setting, the patency rate of iliofemoral bypass can reach over 90% at 5 years, while EV treatment has a patency rate of 72% at 4 years (5).

Nevertheless, OS is associated with higher per-surgical complications and mortality than the EV technique (1). Thus, several EV options have been developed to treat even high-grade lesions, especially in elderly and fragile patients.

In the past years, EV management of CTOs was limited by the impossibility to cross highly calcified vascular segments or by the failure of simple percutaneous transcatheter angioplasties (PTAs). Then, stents able to fix failed PTAs have been commercialized, and percutaneous intentional extraluminal revascularization (PIER) has been described (6) as an ancillary technique, to cross CTOs.

Thus, nowadays, the main cause of technical failure is the inability to re-enter the true lumen (TL) beyond an occluded vascular segment (7), but different devices have been developed to overcome this problem as well.

This review aims to describe different re-entry devices (REDs) developed to increase the success in crossing CTOs of the AT that, otherwise, should be treated surgically or not treated at all.

## Devices and Utilization

### Pioneer Catheter

The first RED was commercialized in 2002, named CrossPoint TransAccess Catheter (TransSonic Systems, Inc., Ithaca, NY) (8) and then Pioneer catheter, after being acquired by Medtronic, Inc. (Santa Rosa, CA).

It consists of a monorail double-lumen catheter, integrated with an intravascular ultrasound (IVUS) array, transducing at 20 MHz [Volcano In-vision Gold IVUS console (Volcano, San Diego, CA)] and equipped with a curved needle allowing for passage of a 0.014″ non-hydrophilic guidewire.

Once the 0.035″ guidewire penetrates the subintimal space, it is exchanged for a 0.014″ guidewire and the Pioneer can be charged on it.

Once the catheter is in place, the IVUS system is activated to detect the position of the TL and the quality of the blood flow, through a color-flow mode (ChromaFlo feature), in order to choose the best area to cross back the intimal layer.

**Figure 1:**
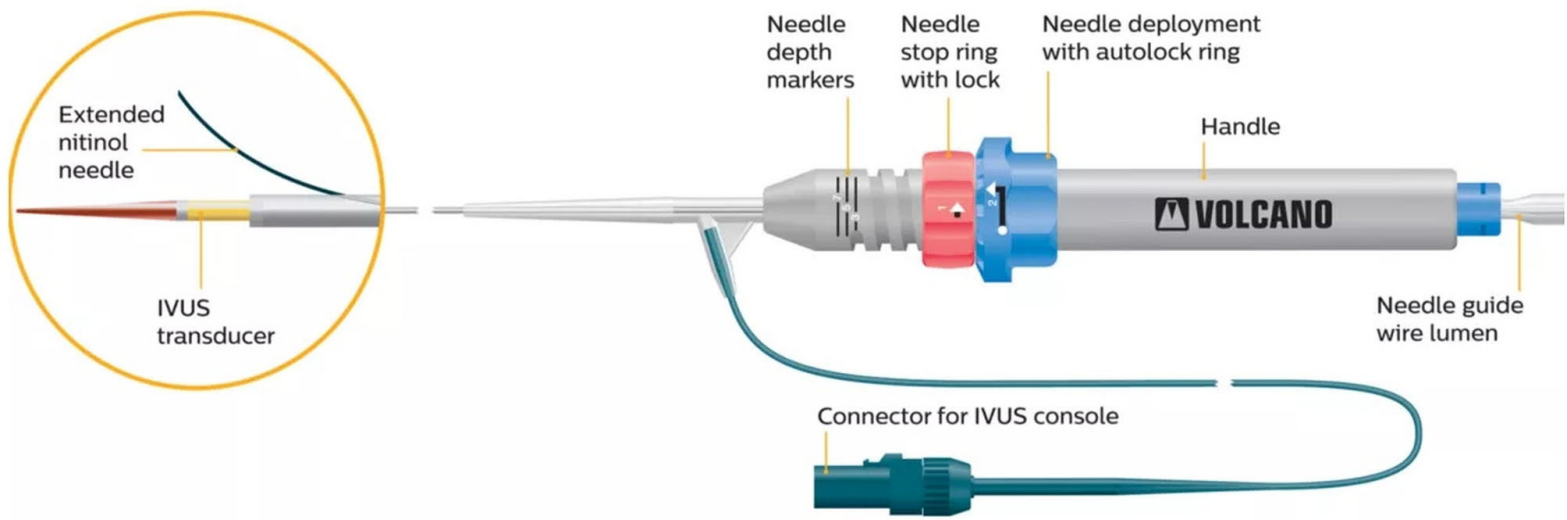
The Pioneer catheter (image courtesy of Philips®), consisting of a monorail double-lumen catheter, integrated with an intravascular ultrasound tip. The handlebar at the bottom of the catheter allows one to push out the lateral needle, located 7 mm below the IVUS array, aiming to perforate the intimal layer and to pass a 0.014″ guidewire into the true lumen.

Then, the operator turns the handlebar at the bottom of the catheter to push out the needle, which is located 7 mm below the IVUS array, in order to perforate the intimal layer and to pass the re-entry guidewire into the TL (Figure 1).

As the trajectory of the needle cannot be modified, the system has to be rotated until the TL is visualized at 12 o'clock in the IVUS cross-sectional still frame, as the needle will come out on that side.

To guarantee a precise puncture of the intimal layer, the length of the needle can be modified through the handlebar of the catheter, with a range varying from 1 to 7 mm.

The first Pioneer device was a 7-F catheter, able to penetrate thick and calcified plaques, but the stiffness of the system made it hard to navigate through bifurcations and densely calcified femoral vessels.

Then, a 6 Fr version of the catheter was released, allowing an easier navigation through narrower vessels.

The tracking system and the high penetration capability of the catheter allows precise iliac or aortic recanalization, reducing the risk for multiple extravascular punctures in the retroperitoneal space (2).

### Outback Catheter

The first Outback catheter was released in 2003 by LuMend (Redwood City, CA). It was in a 5 Fr angled catheter, integrated with a curved needle at its top. The needle could be advanced by pushing it from the bottom of the catheter, to penetrate the intimal layer and to pass a 0.014″ guidewire in the TL. The only way to orientate the puncture was by rotating the catheter and following the needle's direction on the fluoroscopy images (9).

Then, Cordis (Cordis Corporation, Bridgewater, NJ) purchased the device and released a second generation of 6 Fr monorail single-lumen catheters, called Outback LTD, adding a radiopaque marker to detect the position of the catheter on fluoroscopy.

**Figure 2:**
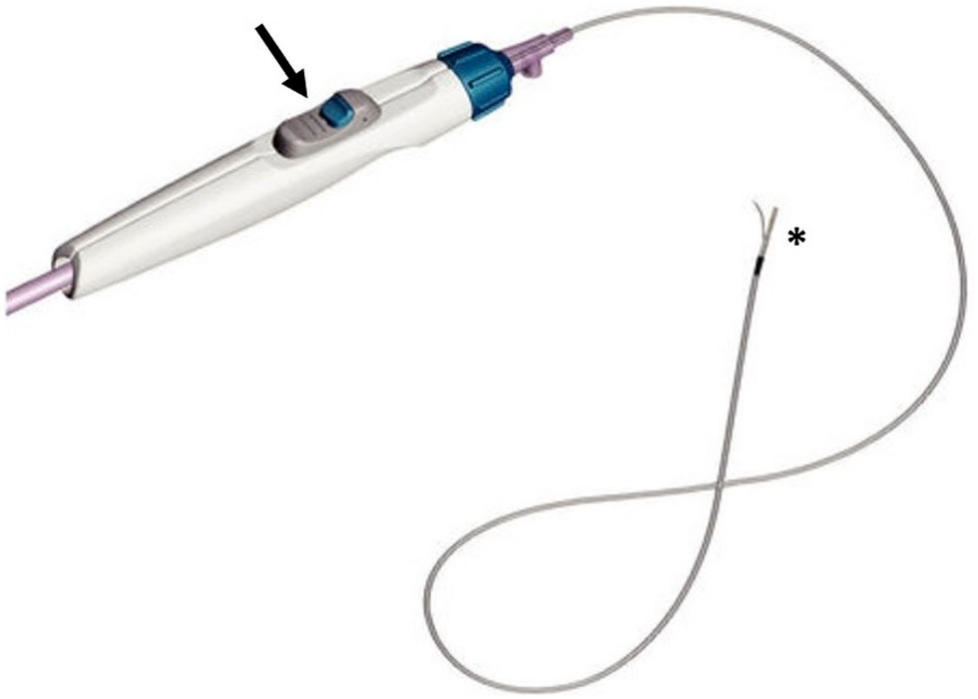
The Outback catheter (image courtesy of Cordis®), consisting of a lateral needle at the top (asterisk), that can be opened thanks to a knob located on the handlebar (arrow).

**Figure 3:**
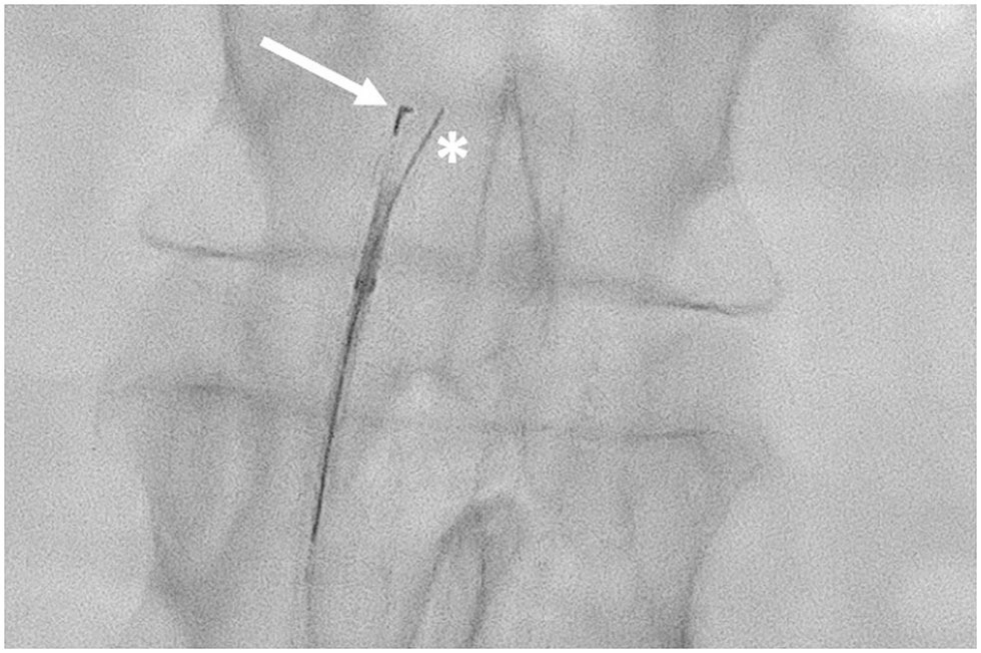
Outback LDT in the subintimal space of the abdominal aorta. The “L” marker is pointed at the true lumen of the aorta (arrow) with the 0.014″ guidewire passing through the needle inner lumen (asterisk).

The needle is placed 12 mm below the tip, while the marker surrounds the top of the catheter and it can have a “T” or an “L” shape on the fluoroscopy images, depending on whether the needle is frontal or lateral to the panel. Thus, by evaluating the aspect of the marker, the operator can detect the position of the needle in respect of the lumen of the artery (Figures 2, 3).

Once the catheter is properly located, the guidewire has to be retracted about 5 cm, and then the needle can be opened (up to 10 mm) thanks to a knob located on the handlebar, and the same guidewire can be advanced in the inner lumen of the needle, to pass in the TL of the target artery.

Because the Outback catheter costs less than the Pioneer one (10), it is often the first choice when a RED is needed.

As for Pioneer, Outback LDT is hard to pass through highly calcified lesions or through tortuous aortic bifurcations. In those cases, the rotation of the RED can cause misplacement of the needle or mismatch between the needle's exit point and the radiological markers, with subsequent unsuccessful puncture (11).

### Offroad Catheter

This device, purchased by Boston Scientific Corporation (Natick, MA) in 2011, gained FDA approval in 2013. It is composed of a needle hypotube and a tipless semicompliant 5.4-mm balloon catheter, compatible with a 0.035″ guidewire.

Once the guidewire is in the subintimal space, the catheter is inserted, and the balloon is inflated, when located beyond the distal part of the CTO.

**Figure 4:**
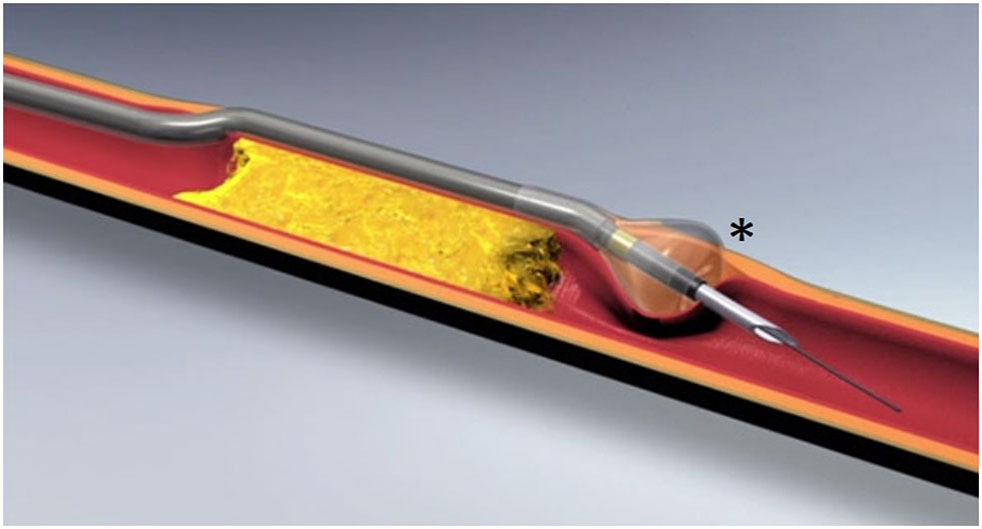
Offroad catheter (image courtesy of Boston Scientific®), composed of an inner lumen, compatible with a 0.035″ guidewire, and a tipless semicompliant balloon catheter with a conical shape (asterisk).

Since the balloon has a conical shape, and the intima is softer than the media and adventitia, the inflation tends to deflect the balloon toward the intima. Then, the needle hypotube is advanced though the intimal layer and a 0.014″ guidewire is passed in the TL (Figure 4).

The inflation system and the shape of the balloon allow one to direct the puncture at the middle of the distal TL and reduce the maneuvers needed to trace the position of the catheter. Then, it exists in two different working lengths (70 and 100 cm), allowing anterograde and retrograde approach to the lesion.

The system is softer than the others previously described and can be helpful when dealing with complex anatomies, but it can fail to penetrate severe calcifications. This is probably the main limitation to its use in the AT.

Some studies came out regarding Offroad utilization in the lower limb territory, but only few cases regarding the AT have been described so far (12).

### Enteer Catheter

**Figure 5:**
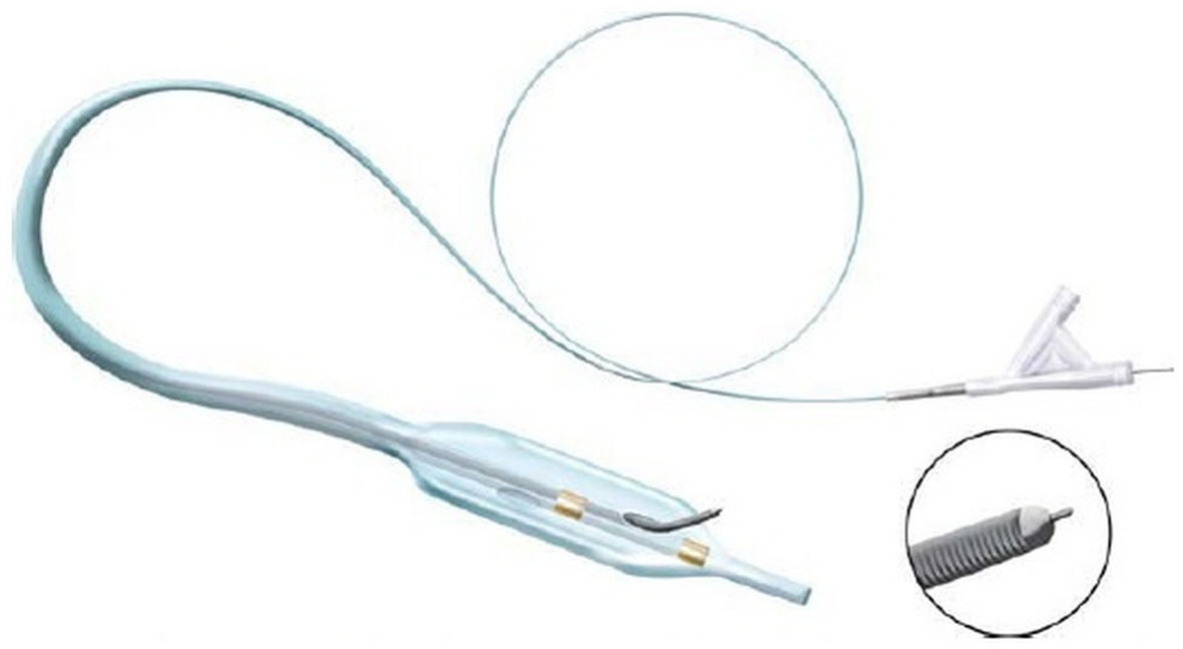
Enteer catheter [image courtesy of Whitlow et al. (13)] has a flat balloon (10 mm long, 2.5 mm wide) at its top provided with a side hole allowing the passage for a 0.014″ guidewire, directed through the intimal layer.

The device has been released in 2012 by Covidien (Mansfield, MA), and it is mainly used to perform coronary angioplasties (13). A flat balloon is integrated at the top of the catheter. Once the balloon is inflated in the subintimal plane, it orientates with the flat side at the interface between the intimal layer and the adventitia. Thus, a stiff 0.014″ guidewire can be inserted through a side-hole placed on the flat part of the balloon to reach the TL (Figure 5).

The catheter is designed to navigate small vessels, and some series reported good results in the coronary and limb territory but, to our best knowledge, no studies on the aortoiliac territory have been performed.

## Discussion

Traditional intraluminal revascularization (IR) is often considered the best option when dealing with stenoses or occlusions of the AT, as it is the most anatomical approach (14). Nevertheless, IR fails in about 20% of cases, depending on the length and strength of the target lesions (15). In those cases, PIER can be a good alternative. The technique was firstly described by Bolia et al. widening the EV approach to patients that could be only treated with OS until then (6).

Traditional PIER, performed with a hydrophilic guidewire and a support catheter, can cross the lesion by dissecting the subintimal space, passing through the occluded area and crossing back distally in the TL (14). However, it fails in revascularizing long or highly calcified iliac CTOs in about 20–25% of cases (2, 16), because of failure to re-enter the TL (17–20) or because the dissection occludes vessels that should be spared to keep patency of the collateral network (6, 18, 21, 22).

RED allows to cross back on a given point of the TL, in short time and without extension of the dissected segment (18, 23), increasing the chance of success as well as reducing procedural time, radiation exposure, and risk of complication related to extensive dissection (7, 24).

**Figure 6:**
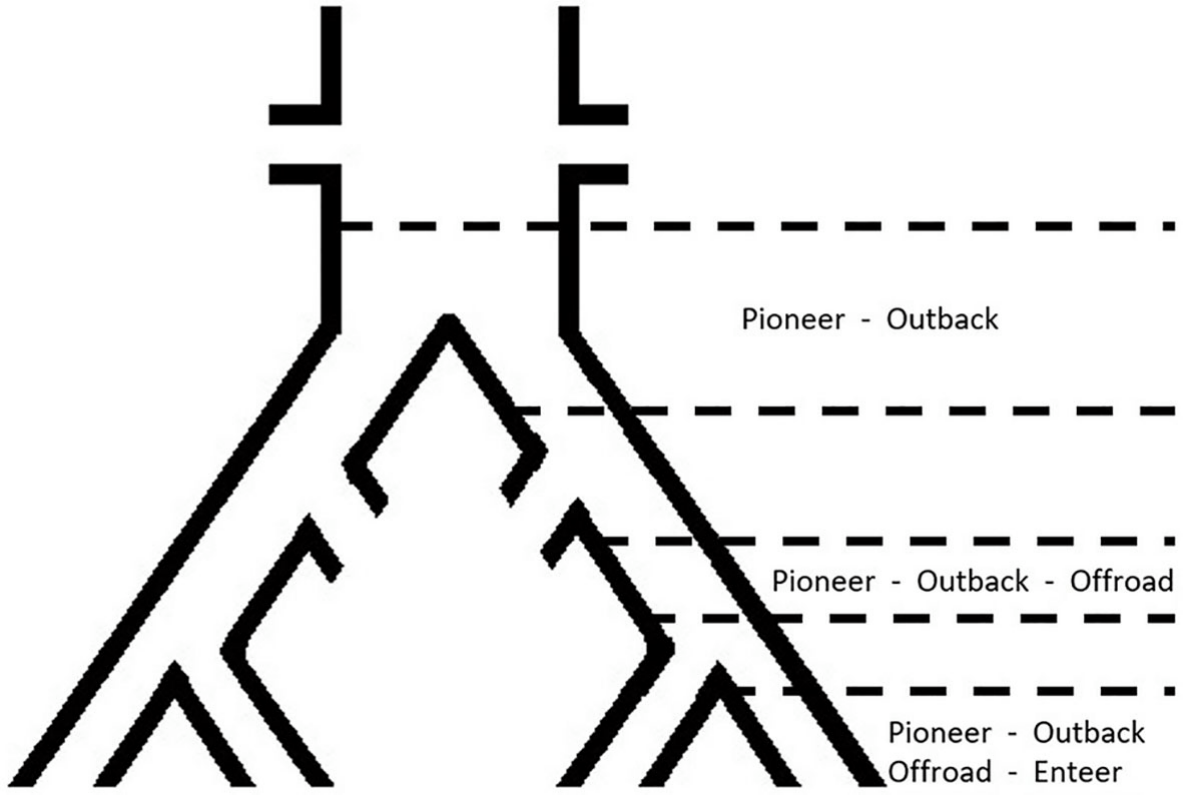
Anatomical diagram of preferred territory of intervention for each re-entry catheter.

The approach at our Institution is to try at first for a traditional PIER and, when unsuccessful attempts last for 10 to 20 min, a RED is inserted and, as reported in literature, it usually solves the cross back in <10 min (7, 25). Our experience in the aortoiliac district is limited to the Outback catheter, even though the Pioneer catheter could be a viable alternative while, in the opinion of the authors, the Enteer and Offroad catheters should be limited to the lower-limb territory, because of their characteristics and size (Figure 6).

Considering the stiffness of the system, the preferred approach is the retrograde homolateral one even though anterograde contralateral approach has been described (2) but, as already said, tortuous bifurcations can misalign inner lumen and markers of the catheter, with subsequent unsuccessful performance (11).

Recently, Kokkinidis et al. (2) published a systematic review of the literature, concerning 1002 patients (and 1112 lesions) who benefit from a PIER of the AT with or without RED. Specifically, 22% of lesions were treated with RED and, out of the eight studies providing the information, RED was used as a first option (without previously attempting traditional PIER) in 50% of papers. In fact, thanks to the possibility to precisely predict the landing zone of the cross back, RED can avoid risky maneuvers that could damage the ostium of important collateral arteries such as hypogastric, sacral, or lumbar ones. However, considering the whole populations, success rate was 89 and 85% after traditional and RED PIER, respectively, while considering only papers that reported the success rate of PIER with RED after failed traditional PIER, it was 100% in four cases and 91% in the last one. The most frequent reason of a failed cross back with RED is the presence of highly calcified landing zones that do not allow the needle passage or do prevent proper visualization of the TL through the IVUS images (2).

In the same review, complication rate was 6.9% in the RED group and 6.7% in the traditional PIER group (2), and this is an encouraging result, considering that patients who benefit from RED are usually burdened with more severe disease. Moreover, when details were given, perforations and embolization were more frequent during traditional PIER, confirming that the tracking systems of REDs reduce the risk of untargeted punctures (2).

In 2018, Kokkinidis et al. (26) also published the first paper comparing long-term outcomes after PIER of CTOs of the AT with or without RED and they found non-significant differences among the two groups, as target lesion revascularization rate at 1 and 5 years was 11 and 29% with RED and 9 and 29% with traditional PIER, with a post-angioplasty stenting rate of 82% after RED and 87% after traditional PIER.

The main limitation of REDs is their cost, that is, $1800 and $3100 for Outback LTD and Pioneer, respectively (10). A study carried out on the US market found that, even considering the secondary medical expenses of an unsuccessful revascularization, the Outback catheter increases the cost of the procedure for a mean of $400 (27).

In conclusion, the available literature regarding REDs is encouraging and shows good technical success, allowing access to EV therapies also to those patients suffering from severe vascular diseases that, for decades, were not even imaginable to treat with non-invasive approaches.

## Author Contributions

HK conceived the review. LP wrote the manuscript. HK, VT, and LP contributed to the discussion.

## Conflict of Interest

The authors declare that the research was conducted in the absence of any commercial or financial relationships that could be construed as a potential conflict of interest.
